# Assessment of the toxicogenic effects and cell death potential of the ester (*Z*)-methyl 4-((1,5-dimethyl-3-oxo-2-phenyl-2,3-dihydro-1*H*-pyrazol-4-yl)amino)-4-oxobut-2-anoate in combination with cisplatin, cyclophosphamide and doxorubicin

**DOI:** 10.1590/1678-4685-GMB-2017-0279

**Published:** 2019-06-27

**Authors:** Rodrigo Juliano Oliveira, Fabrícia Paniago Ajala Nery Pereira, Ingridhy Ostaciana Maia Freitas da Silveira, Ricardo Vieira de Lima, Claudia Rodrigues Berno, João Renato Pesarini, Andréia Conceição Milan Brochado Antoniolli-Silva, Antônio Carlos Duenhas Monreal, Beatriz Adilson, Dênis Pires de Lima, Roberto da Silva Gomes

**Affiliations:** 1 Centro de Estudos em Célula Tronco, Terapia Celular e Genética Toxicológica, Hospital Universitário “Maria Aparecida Pedrossian”, Empresa Brasileira de Serviços Hospitalares, Campo Grande, MS, Brazil; 2 Programa de Mestrado em Farmácia, Centro de Ciências Biológicas e da Saúde, Universidade Federal de Mato Grosso do Sul, Campo Grande, MS, Brazil; 3 Programa de Pós-graduação em Saúde e Desenvolvimento na Região Centro-Oeste, Faculdade de Medicina “Dr. Hélio Mandetta”, Universidade Federal de Mato Grosso do Sul, Campo Grande, MS, Brazil; 4 Programa de Pós-graduação em Genética e Biologia Molecular, Centro de Ciências Biológicas, Universidade Estadual de Londrina, Londrina, PR, Brazil; 5 Programa de Pós-graduação em Química, Instituto de Química, Universidade Federal de Mato Grosso do Sul, Campo Grande, MS, Brazil; 6 Laboratório de Síntese e Modificação Molecular, Faculdade de Ciências Exatas e Tecnologias, Universidade Federal da Grande Dourados, Dourados, MS, Brazil; 7 Graduação em Biomedicina, Universidade Católica Dom Bosco, Campo Grande, MS, Brazil; 8 Mestrado em Ciências Veterinárias, Faculdade de Medicina Veterinária e Zootecnia, Universidade Federal de Mato Grosso do Sul, Campo Grande, MS, Brazil; 9 Chemistry and Chemical Biology Department, Harvard University, Cambridge, MA, USA.

**Keywords:** Cancer, 4-aminoantipyrine, 1,4-dioxo-butenyl, chemoprevention, chemotherapy

## Abstract

Despite rapid advances in both the early detection and treatment of cancer, the mortality from this disease remains high, which justifies the development of new products that are more selective and effective and have fewer side effects. Accordingly, a novel ester was synthesized that contains two pharmacophores with important biological activities: (I) 4-aminoantipyrine, which has anti-inflammatory and antioxidant effects, and (II) the pharmacophore 1,4-dioxo-butenyl, which has cytotoxic activity. When administered alone, this compound is non-genotoxic, and it does not cause an increasing in splenic phagocytosis. Nevertheless, it can induce cell death. When administered in combination with commercial chemotherapeutic agents, such as doxorubicin, cisplatin, and cyclophosphamide, the ester shows antigenotoxic activity and decreases phagocytosis and reduces the potential to cause cell death. These results indicate that the compound should not be used in combination with chemotherapeutic agents that exert their effect through DNA damage, an important feature of antitumor drugs.

## Introduction

Despite advances in the early diagnosis and treatment of cancer, this disease is the leading cause of death in developed countries and the second main cause of death in developing countries (Umar *et al.*, 2012; Reimers *et al.*, 2014). This lethality is due to the high complexity of the disease, the high degree of heterogeneity of tumor cells (Chen and Wang, 2016) and the capacity for uncontrolled proliferation (Helleday *et al.*, 2008).

Chemotherapeutic agents are the main treatment strategy against cancer and generally act by causing DNA damage that induces cell-cycle arrest and programmed cell death (Helleday *et al.*, 2008; Reimers *et al.*, 2014). However, they are poorly selective and, therefore, induce DNA damage in healthy cells, in addition to causing several other side effects (Navarro *et al.*, 2014; Carvalho *et al.*, 2015; Oliveira *et al.*, 2015). The treatment efficiency also varies depending on the type of cancer (Chabner and Roberts, 2005; Vichaya *et al.*, 2015). As new drugs must be developed that are more effective and selective and exhibit fewer side effects, there is a growing interest in the synthesis of drugs that are designed specifically for this purpose.

Antipyrines are possible starting points for the synthesis of antitumor molecules (Khanduja *et al.*, 1984; Nishio *et al.*, 2005; Berno *et al.*, 2016; Oliveira *et al.*, 2018). In the present study, 4-aminoantipyrine was used (Jain *et al.*, 2006) due to its antioxidant (Teng and Liu, 2013; Ghorab *et al.*, 2014), analgesic (Costa *et al.*, 2006), anti-inflammatory (Burdulene *et al.*, 1999), antipyretic, and antiviral (Evstropov *et al.*, 1992) effects.

Several previous studies of biological activity indicated that the addition of 4-aminoantipyrine to a chemical structure can enhance its biological response (Teng and Liu, 2013; Ghorab *et al.*, 2014; Aly *et al.*, 2011; Oliveira *et al.*, 2018). Thus, the ester (*Z*)-methyl 4-((1,5-dimethyl-3-oxo-2-phenyl-2,3-dihydro-1*H*-pyrazol-4-yl)amino)-4-oxobut-2-enoate (IR-04) was synthesized from 4-aminoantipyrine via the carbonyl addition of a maleimide (1,4-dioxo-2-butenyl). The 1,4-dioxo-butenyl portion was added due to its strong cytotoxic effect, a key feature when synthesizing DNA damage inducers and searching for possible candidates for new antitumor drugs (Jha *et al.*, 2010).

In general, the structure of IR-04 comprises a region (4-aminoantipyrine) that has several biologically active sites and another region (1,4-dioxo-2-butenyl moiety) with anticancer activity. Furthermore, this ester could potentially modulate plasma membrane polarity and, thus, facilitate access to the cell interior (Jha *et al.*, 2010). These properties have guided the design and synthesis of this ester, which was evaluated for its genotoxic, phagocytic and cell death potentials, as well as its effects when combined with doxorubicin, cisplatin and cyclophosphamide.

## Material and Methods

### Chemistry

All reagents and solvents for synthesis and NMR measurements were purchased commercially and used without further purification. The microwave reaction was carried out in a Discover-CEM Microwave Reactor. ^1^H and ^13^C NMR spectra were recorded at room temperature on a Varian-DPX-300 (10% in DMSO-*d*_*6*_ and CDCl_3_ solutions at 298Kfor the acid and the ester, respectively) operating at 300.132 MHz for ^1^H measurements and 75.476 MHz for ^13^C measurements. Data processing was carried out on a Solaris workstation. The ^1^H and ^13^C chemical shifts are given on the δ scale (ppm) and were referenced to internal tetramethylsilane (TMS); coupling constants *J* are reported in hertz (Hz). The abbreviations s, d and m were used for simplet, douplet, and multiplet, respectively.

#### Synthesis of (Z)-4-((1,5-dimethyl-3-oxo-2-phenyl-2,3 dihydro-1H-pyrazol-4-yl) amino)-4-oxobut-2-enoic acid (IR-01)

IR-01 was synthesized according to Oliveira *et al.* (2018). Briefly, a sealed tube coupled to a microwave reactor containing 4-aminoantipyrine (10.20 mmol) and maleic anhydride (10.20 mmol) was subjected to 150 W microwave irradiation at 90 °C for 10 s. Then, the tube was cooled and the product was recrystallized with ethyl acetate. Yield: 93%.

**Figure 1:**
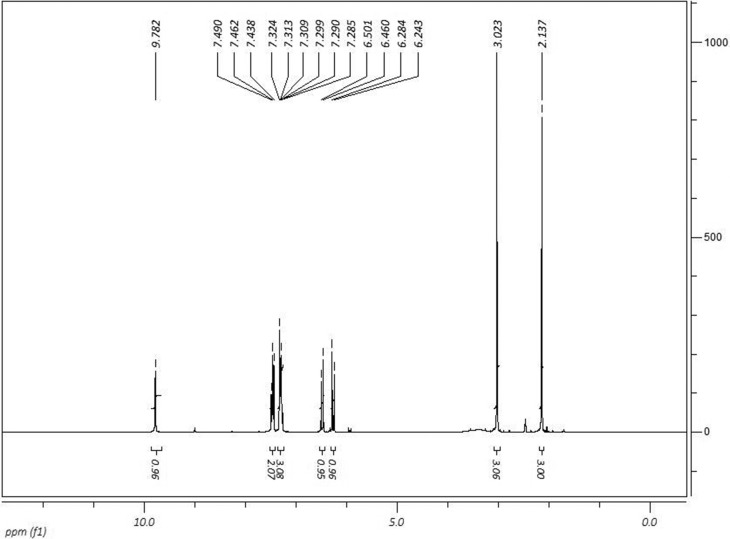
^1^H NMR spectra of (*Z*)-4-((1,5-dimethyl-3-oxo-2-phenyl- 2,3dihydro-1*H*-pyrazol-4-yl) amino)-4-oxobut-2-enoic acid (IR-01) in DMSO-*D*_*6*_ at 300 MHz.

**Figure 2:**
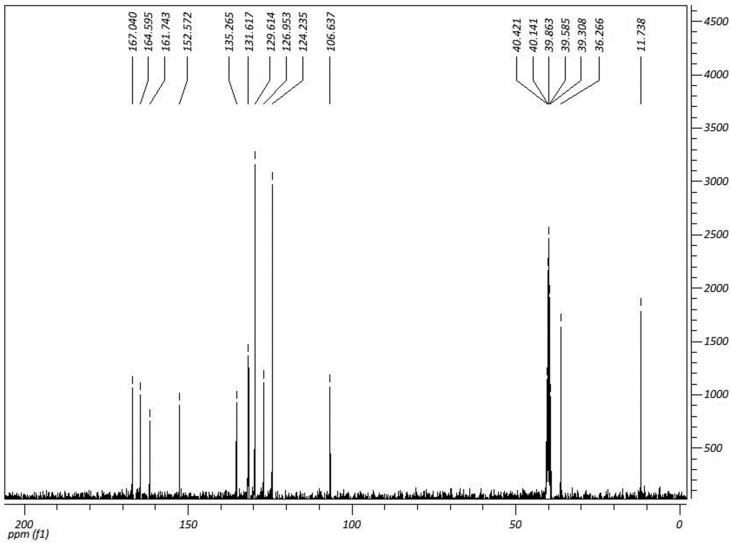
^13^C NMR spectra of (*Z*)-4-((1,5-dimethyl-3-oxo-2-phenyl- 2,3dihydro-1*H*-pyrazol-4-yl) amino)-4-oxobut-2-enoic acid (IR-01) in DMSO-*D*_*6*_ at 75 MHz.

^1^H NMR (DMSO-*d*_*6*_, 300 MHz) δ (ppm): 2.13 (s, 3H); 3.02 (s, 3H); 6.26 (d, 1H, *Jcis*= 12.3Hz); 6.48 (d, 1H, *Jcis*= 12.3Hz); 7.30 (m, 3H); 7.46 (m, 2H); 9.78 (s, 1H). ^13^C NMR (DMSO-*d*_*6*_, 75 MHz) δ (ppm): 11.73 (CH_3_); 36.26 (CH_3_); 106.63 (C); 124.23 (CH); 126.95 (CH); 129.95 (CH); (CH); 131.61 (CH); 135.26 (C); 152.57 (C); 161.74 (C=O); 164.59 (C=O); 167.04 (C=O). ^1^H and ^13^C NMR spectra are shown in Figures 1 and 2.

#### Synthesis of the ester (Z)-methyl 4-((1,5-dimethyl-3-oxo-2-phenyl-2,3-dihydro-1H-pyrazol-4-yl)amino)-4-oxobut-2- enoate (IR-04)

In a 50 mL round-bottom flask coupled to a Dean-Stark trap, 2.70 g (8.9 mmol) of IR-01 acid, 25 mL of methanol and 2.5 mL of sulfuric acid were added. The reaction was refluxed for 3 hours. Then, the reaction mixture was washed with saturated sodium bicarbonate (2x30 mL), water (1x30 mL) and ethyl acetate (2x20 mL). The organic phase was dried over MgSO_4_ and concentrated by reduced pressure. The product was purified by chromatographic column using hexane/ethyl acetate (1:9) as eluent. Yield: 77%.

**Figure 3:**
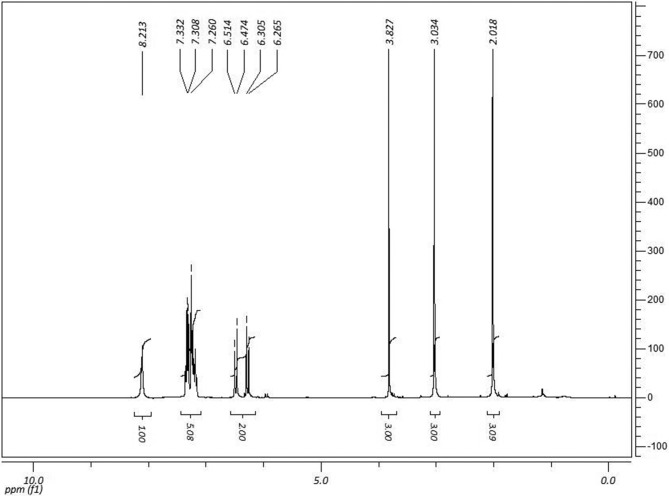
^1^H NMR spectra of (*Z*)-methyl 4-((1,5-dimethyl-3-oxo-2-phenyl-2,3-dihydro-1*H*-pyrazol-4-yl)amino)-4-oxobut-2-anoate (IR-04) in DMSO-*D*_*6*_ at 300 MHz.

**Figure 4:**
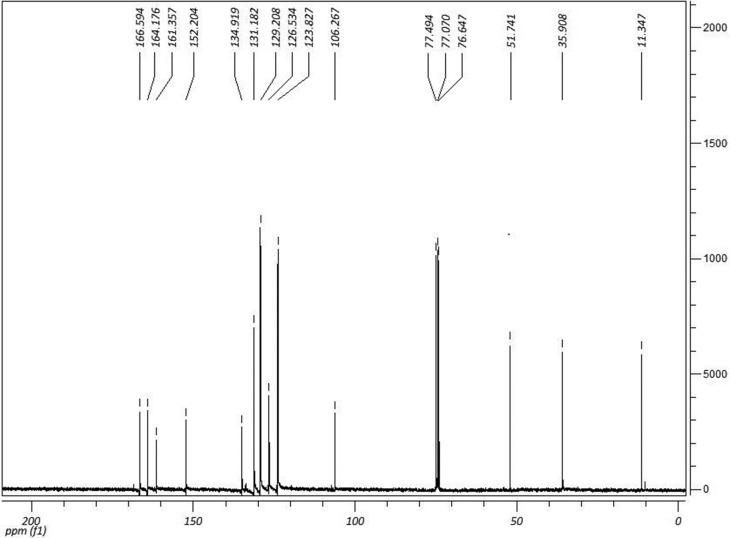
^13^C NMR spectra of (*Z*)-methyl 4-((1,5-dimethyl-3-oxo-2-phenyl-2,3-dihydro-1*H*-pyrazol-4-yl)amino)-4-oxobut-2-anoate (IR-04) in DMSO-*D*_*6*_ at 75 MHz.

^1^H NMR (CDCl_3_, 300 MHz) δ (ppm): 2.01 (s, 3H); 3.03 (s, 3H); 3.82 (s, 3H); 6.28 (d, 1H, *Jcis*= 12.3Hz); 6.49 (d, 1H, *Jcis*= 12.3Hz); 7.30 (m, 5H); 8.21 (s, 1H). ^13^C NMR (CDCl_3_, 75 MHz) δ (ppm): 11.34 (CH_3_); 35.90 (CH_3_); 51.74 (CH_3_); 106.26 (C); 123.82 (CH); 126.53 (CH); 129.20 (CH); 131.18 (CH); 134.91 (C); 152.20 (C); 161.35 (C=O); 164.17 (C=O); 166.59 (C=O). ^1^H and ^13^C NMR spectra are shown in Figures 3 and 4.

### Chemical agents, animals and experimental design

The chemotherapeutic agents were obtained commercially and administered in a single dose that was previously determined in pilot experiments (data not shown). Doxorubicin (Glenmark Pharmaceuticals Ltd., Argentina. MS Reg. No. 1.1013.0232.002-4, Lot #21130040) was diluted in distilled water and administered at a dose of 16 mg/kg body weight (b.w.) intraperitoneally (i.p.). Cisplatin (Accord Pharmaceuticals Ltd., UK. MS Reg. No. 1.5537.0002.003-7, Lot #88549) was administered at a dose of 6 mg/kg (b.w., i.p.). Cyclophosphamide (Genuxal^®^, Baxter Ltd., Germany. MS Reg. No. 1.00683.0168.003-1, Lot #F728) was diluted in distilled water and administered at a dose of 100 mg/kg (b.w., i.p.).

A total of 80 male adult (8-10 weeks old) mice (Swiss) with an average weight of 35 g from the State Department of Animal and Plant Health (Agência Estadual de Defesa Sanitária Animal e Vegetal - IAGRO) were kept in polypropylene boxes covered with sawdust in ventilated racks (ALESCO®) under controlled climate and light conditions (12 hours of light and 12 hours of dark, temperature of approximately 22 ± 2°C, relative humidity of 5510%), and they had *ad libitum* access to commercial feed (Nuvital, Nuvilab®) and filtered water.

**Figure 5:**
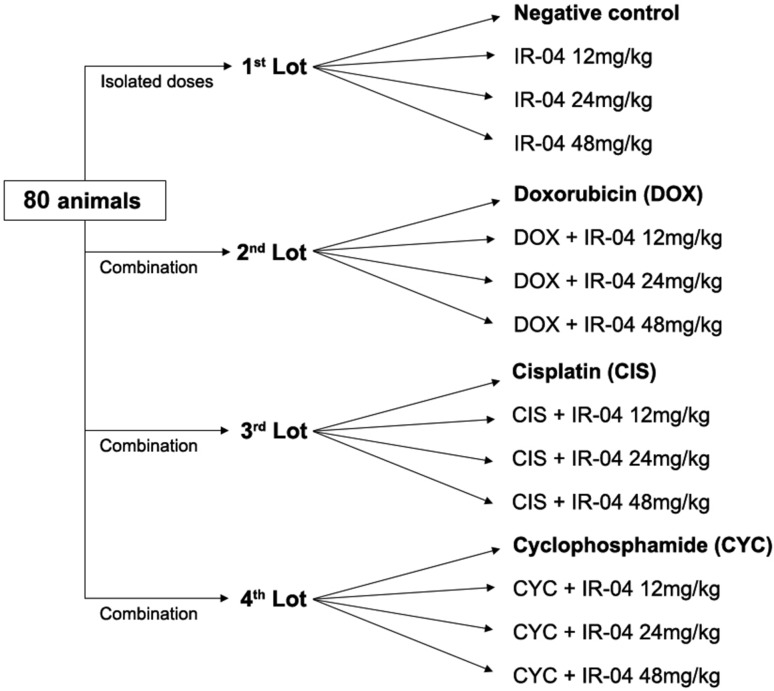
Experimental design (doses and groups).

The animals were divided into four lots of 20 animals each. The first lot was used for genotoxicity assessment and was divided into 4 groups (n=5) as follows: negative control, IR-04 12, 24 and 48 mg/kg. The other three lots were used for antigenotoxicity assays with doxorubicin (n=5), cisplatin (n=5) and cyclophosphamide (n=5) as positive controls and were designed to have 3 combination groups (n=5) with IR-04 doses of 12, 24 and 48 mg/kg associated with their respective positive control (Figure 5). After the experiments, the animals were euthanized by cervical dislocation for the collection of biological materials.

The study was conducted according to the guidelines of the Universal Declaration of Animal Rights. The study protocol was approved by the Ethics Committee on Animal Use of the Federal University of Mato Grosso do Sul-UFMS (protocol number 399/2012).

### Biological assays

#### Peripheral blood Comet assay

A peripheral blood sample collected 24 hours after treatment was used to perform the Comet assay according to the protocol established by Singh *et al.* (1988) with modifications by Carvalho *et al.* (2015). In total, 100 cells/animal were visually analyzed under a fluorescence microscope (BIOVAL®, L2000A Model) with a 40x objective, 420-490 nm excitation filter and 520 nm barrier filter.

#### Peripheral blood Micronucleus assay

Peripheral blood samples were collected 24, 48 and 72 hours after treatment for the Micronucleus assay, according to the protocol previously described by Hayashi *et al.* (1990), with modifications by Carvalho *et al.* (2015). Approximately 20 μL of peripheral blood was collected and placed on a microscope slide previously covered with 20 μL of acridine orange (1 mg/mL). The slides were kept in a freezer (-20 °C), and subsequently, 2,000 cells/animal were analyzed under a fluorescence microscope (BIOVAL®, L Model 2000A) with a 40x objective, 420-490 nm excitation filter and 520 nm barrier filter to count the number of micronuclei.

#### Cell death assay

Liver and kidney tissues were collected and macerated in physiological solution, and 100 μL of this suspension was used for the smear preparation. After the slides were dried, they were fixed in Carnoy’s solution for 5 min, subsequently immersed in different concentrations of ethanol (95%, 75%, 50% and 25%), washed in McIlvaine buffer for 5 min, stained with acridine orange (1 mg/mL for 5 min) and washed again with McIlvaine buffer. The analysis was performed under a fluorescence microscope (BIOVAL® Model L 200A) with a 40x objective, 420-490 nm excitation filter and 520 nm barrier filter. The cells were classified based on DNA fragmentation patterns, and 200 cells/animal were counted (Navarro *et al.*, 2014; Carvalho *et al.*, 2015; Oliveira *et al.*, 2015).

#### Splenic phagocytosis assay

Approximately 1/3 of the spleen was macerated in saline solution. Then, 100 μL of the cell suspension was placed on a slide previously stained with 20 μL of acridine orange (1 mg/mL) and covered with a coverslip (Hayashi *et al.*, 1990). The analysis was performed under a fluorescence microscope with a 40x objective, 420-490 nm excitation filter and 520 nm barrier filter. For this assay, 200 cells/animal were analyzed for the presence or absence of phagocytosis, as described by Carvalho *et al.* (2015).

Percentage points were used to calculate the extent of phagocytosis reduction. For this purpose, the frequency of cells that exhibited evidence of phagocytosis in the positive control groups (doxorubicin, cisplatin or cyclophosphamide) was considered 100%, and the percentage for each associated group was calculated by the rule of three. This calculated value was then subtracted from 100, and the result is presented as the reduction in phagocytic activity in percentage points.

#### Calculation of percent damage reduction (%DR)

The damage reduction percentage is used to assess the chemopreventive capacity of a substance when combined with a known DNA damage inducer. For this purpose, the formula proposed by Manoharan and Banerjee (1985) and Waters *et al.* (1990) was used:

$$\%\text{DR}=\left(\frac{\text{Mean of Positive Control}-\text{Mean of Associated Group}}{\text{Mean of Positive Control}-\text{Mean of Control}}\right)\times 100$$

#### Statistical analysis

The values are expressed as the mean ± standard error of the mean (SE) or percentage. Parametric data were analyzed by Student’s *t*-test, according to the nature and distribution of the data (GraphPad Prism, Version 3.2, Graph-Pad Software Inc., San Diego, CA, USA). The significance level adopted was *p* < 0.05.

## Results

### Synthesis

**Figure 6:**
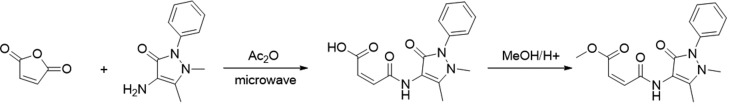
Synthesis of ester (*Z*)-methyl 4-((1,5-dimethyl-3-oxo-2-phenyl-2,3-dihydro-1*H*-pyrazol-4-yl)amino)-4-oxobut-2-anoate.

The synthesis of ester IR-04 was performed in only two steps, with 74.6% overall yield (Figure 6). The use of a microwave reactor helped to decrease the use of solvents and optimized the reaction time.

The formed products were characterized by ^1^H and ^13^C NMR analysis with deuterated dimethyl sulfoxide for IR-01 sample and deuterated chloroform in the IR-04 sample with TMS as internal reference. Chemical shifts and integrations consistent with the proposed molecules were observed.

In the ^1^H-NMR spectra (Figure 3), the formation of IR-04 was indicated by the simplet that was proportional to three hydrogens of the methoxy group at 3.60 ppm. Furthermore, a methoxyl group signal was also observed in the ^13^C NMR spectra (Figure 4) at 51.8 ppm. Both signals are consistent with the replacement of a hydroxyl group by a methoxy group.

All other signals also showed chemical shifts and integrations related to the IR-04 ester molecule.

### Biological assays

#### Comet assay

The results showed that none of the three tested doses of IR-04 had genotoxic activity, and all were able to reduce the extent of basal damage. When combined with the chemotherapeutic agents, IR-04 showed antigenotoxic activity, and the damage reduction percentages ranged from 110.98% to 131.81% for doxorubicin, from 136.40% to 138.94% for cisplatin, and 96.29% to 111.11% for cyclophosphamide (Table 1).

**Table 1:** Means ± SE of damaged cells, distribution between damage classes, and scores related to genotoxicity and anti-genotoxicity tests of IR-04 by means of the comet assay.

Experimental Groups	Damaged cells	Classes				Score	%DR
		0	1	2	3		
LOT 1							
NC	23.75 ± 2.39	76.25 ± 2.39	23.75 ± 2.39	0.00 ± 0.00	0.00 ± 0.00	23.75 ± 2.39ª^*^	
IR-04 12mg/kg	10.80 ± 0.37^a*^	87.20 ± 2.31ª*	1.80 ± 0.37ª^*^	0.00 ± 0.00^a^	0.00 ± 0.00	10.80 ± 0.37ª^*^	
IR-04 24mg/kg	9.20 ± 1.64^a*^	88.80 ± 2.31ª*	8.80 ± 0.58ª^*^	0.00 ± 0.00^a^	0.00 ± 0.00	9.20 ± 0.73ª^*^	
IR-04 48mg/kg	2.50 ± 0.86^a*^	97.50 ± 0.86ª*	2.50 ± 0.67ª^*^	0.00 ± 0.00^a^	0.00 ± 0.00	2.50 ± 0.86ª^*^	
LOT 2							
DOX	71.80 ± 2.03^a^	28.40 ± 2.08^a^	67.40 ± 2.08^a^	4.40 ± 0.24^a*^	0.00 ± 0.00	76.20 ± 2.01^a^	
+ IR-04 12mg/kg	3.60 ± 0.40^b*^	96.20 ± 0.58^b*^	3.60 ± 0.40^b*^	0.00 ± 0.00^b^	0.00 ± 0.00	3.06 ± 0.40^b*^	129.16
+ IR-04 24mg/kg	2.20 ± 0.37^b*^	97.80 ± 0.37^b*^	2.20 ± 0.37^b*^	0.00 ± 0.00^b^	0.00 ± 0.00	2.20 ± 0.37^b*^	131.81
+ IR-04 48mg/kg	16.50 ± 1.19^b*^	83.50 ± 1.19^b*^	16.50 ± 1.19^b*^	0.00 ± 0.00^b*^	0.00 ± 0.00	16.50 ± 1.19^b*^	110.98
LOT 3							
CIS	71.25 ± 1.43^a^	28.75 ± 1.43^b^	23.75 ± 2.39^a^	5.00 ± 0.70^b*^	0.00 ± 0.00	76.25 ± 1.37^a^	
+ IR-04 12mg/kg	5.25 ± 0.25^c*^	94.75 ± 0.25^c*^	5.25 ± 0.25^c*^	0.00 ± 0.00^b*^	0.00 ± 0.00	5.25 ± 0.31^c*^	138.94
+ IR-04 24mg/kg	5.00 ± 0.31^c*^	95.00 ± 0.31^c*^	5.00 ± 0.31^c*^	0.00 ± 0.00^b*^	0.00 ± 0.00	5.00 ± 0.31^c*^	136.40
+ IR-04 48mg/kg	7.25 ± 1.31^c*^	92.75 ± 1.31^c*^	7.25 ± 1.31^c*^	0.00 ± 0.00^b*^	0.00 ± 0.00	7.25 ± 1.31^c*^	137.36
LOT 4							
CYC	77.75 ± 1.54^a^	17.80 ± 4.60^a^	71.50 ± 1.32^a^	6.25 ± 0.25^b*^	0.00 ± 0.00	81.50 ± 3.57^a^	
+ IR-04 12mg/kg	17.75 ± 1.31^d*^	82.25 ± 1.31^d*^	17.75 ± 1.31^d*^	0.00 ± 0.00^b*^	0.00 ± 0.00	17.75 ± 1.31^d*^	111.11
+ IR-04 24mg/kg	16.00 ± 1.31^d*^	84.0 ± 2.62^d*^	5.00 ± 0.31^d*^	1.80 ± 1.11^b*^	0.00 ± 0.00	17.80 ± 3.63^d*^	106.94
+ IR-04 48mg/kg	20.60 ± 1.69^d*^	79.4 ± 1.69^d*^	19.6 ± 1.63^d*^	1.00 ± 1.00^b*^	0.00 ± 0.00	21.6 ± 2.24^d*^	96.29

SE: Standard error of the mean; %DR: Percent damage reduction; NC: Negative control; DOX: Doxorubicin group; CIS: Cisplatin group; CYC: Cyclophosphamide group. ^a^Statistically compared to the NC group; ^b^Statistically compared to the DOX group; ^c^Statistically compared to the CIS group; ^d^Statistically compared to the CYC group; ^*^statistically significant difference (*p*<0.05; ANOVA/*t*-Student).

#### Micronucleus assay

The micronucleus assay suggested that IR-04 did not cause chromosomal damage, and that IR-04 combined with the chemotherapeutic agents doxorubicin and cisplatin prevented chromosomal damage at all dose levels with damage reduction percentages ranging from 26.17 to 80% and from 4.65 to 78.31%, respectively. In combination with cyclophosphamide, there was no chemoprevention 24 hours after the high dose of IR-04 was applied, or 48 hours after the lowest dose and the intermediate dose were applied. The DNA damage reduction percentages ranged from 0.38 to 82.60% for the combination of cyclophosphamide with IR-04 (Table 2).

**Table 2:** Results related to the ability of IR-04 in cause or prevent chromosomal damage through the micronucleus assay.

Experimental Groups	Mean ± SE			% Damage reduction		
	24 h	48 h	72 h	24 h	48 h	72 h
LOT 1						
NC	5.60 ± 1.07	8.40 ± 0.81	8.20 ± 1.59	-	-	-
IR-04 12mg/kg	8.80 ± 0.86ª	7.20 ± 1.06^a^	7.60 ± 1.60^a^	-	-	-
IR-04 24mg/kg	6.75 ± 1.54^a^	7.75 ± 1.25^a^	7.80 ± 1.65^a^	-	-	-
IR-04 48mg/kg	9.20 ± 1.71^a^	9.20 ± 0.86^a^	10.00 ± 0.89^a^	-	-	-
LOT 2						
DOX	35.40 ± 1.43ª^*^	37.00 ± 1.51ª^*^	50.20 ± 2.95ª^*^			
+ IR-04 12mg/kg	27.60 ± 1.40^b*^	21.00 ± 1.58^b*^	20.60 ± 2.69^b*^	26.17	55.94	70.47
+ IR-04 24mg/kg	20.50 ± 0.95^b*^	27.20 ± 2.13^b*^	22.40 ± 2.11^b*^	50	34.26	66.19
+ IR-04 48mg/kg	27.40 ± 1.74^b*^	26.20 ± 1.59^b*^	16.60 ± 1.80^b*^	26.84	37.76	80
LOT 3						
CIS	49.75 ± 2.42ª^*^	51.80 ± 2.63ª^*^	41.60 ± 1.77ª^*^			
+ IR-04 12mg/kg	26.00 ± 1.58^c*^	23.75 ± 1.10^c*^	17.00 ± 1.00^c*^	10.52	4.65	6.02
+ IR-04 24mg/kg	19.25 ± 1.97^c*^	28.20 ± 1.24^c*^	15.00 ± .2.48^c*^	40.13	22.98	18.07
+ IR-04 48mg/kg	23.25 ± 1.79^c*^	15.00 ± 2.48^c*^	10.00 ± 0.91^c*^	22.58	50.31	78.31
LOT 4						
CYC	32.00 ± 1.47^a*^	34.40 ± 1.86ª^*^	22.00 ± 1.47ª^*^			
+ IR-04 12mg/kg	20.60 ± 1.53^d*^	34.50 ± 2.50^d^	14.25 ± 1.37^d*^	43.18	0.38	56.15
+ IR-04 24mg/kg	19.75 ± 1.37^d*^	32.20 ± 1.31^d^	15.25 ± 1.75^d*^	46.40	8.46	48.91
+ IR-04 48mg/kg	30.80 ± 2.03^d^	27.00 ± 1.70^d*^	10.60 ± 1.03^d*^	4.54	28.46	82.60

SE: Standard error of the mean; NC: Negative control; DOX: Doxorubicin group; CIS: Cisplatin group; CYC: Cyclophosphamide group. ^a^Statistically compared to the NC group; ^b^Statistically compared to the DOX group; ^c^Statistically compared to the CIS group; ^d^Statistically compared to the CYC group; ^*^statistically significant difference (*p*<0.05; ANOVA*/t*-Student).

#### Cell death assay

IR-04 induces cell death in liver and kidney cells when administered alone. The increases caused by the 12, 24 and 48 mg/kg doses were respectively 1.87x, 2.63x and 3.30x in the liver, and 1.82x, 3.51x and 2.85x in the kidney. When this same compound was combined with doxorubicin, cisplatin and cyclophosphamide, all doses of the compound, except the highest dose in combination with doxorubicin in the liver, caused less cell death (*p*<0.05) when compared to the chemotherapeutic agent alone (Table 3).

**Table 3:** Cell death evaluation on mice’ kidneys and liver.

Experimental Groups	Liver		Kidneys	
	Number of dead cells	Mean ± SE	Number of dead cells	Mean ± SE
LOT 1				
NC	79	15.80 ± 1.24	73	14.60 ± 0.81
IR-04 12 mg/kg	148	29.60 ± 2.20^a*^	138	26.60 ± 1.24^a*^
IR-04 24 mg/kg	208	41.60 ± 0.81^a*^	208	41.60 ± 0.92^a*^
IR-04 48 mg/kg	261	52.20 ± 1.02^a*^	256	51.20 ± 0.96^a*^
LOT 2				
DOX	550	110.00 ± 0.70^a*^	546	109.20 ± 0.58^a*^
+ IR-04 12 mg/kg	486	97.20 ± 0.37^b*^	481	96.20 ± 0.58^b*^
+ IR-04 24 mg/kg	516	103.20 ± 1.02^b*^	511	102.20 ± 0.86^b*^
+ IR-04 48 mg/kg	540	108.00 ± 0.54^b^	535	107.00 ± 0.54^b*^
LOT 3				
CIS	552	110.40 ± 0.50^a*^	547	109.40 ± 0.67^a*^
+ IR-04 12 mg/kg	472	94.04 ± 0.87^c*^	469	93.80 ± 1.11^c*^
+ IR-04 24 mg/kg	533	106.60 ± 1.20^c*^	522	104.40 ± 1.20^c*^
+ IR-04 48 mg/kg	532	106.40 ± 1.36^c*^	526	105.20 ± 1.65^c*^
LOT 4				
CYC	547	109.40 ± 0.40^a*^	540	108.00 ± 0.54^a*^
+ IR-04 12 mg/kg	474	94.80 ± 0.86^d*^	472	94.40 ± 1.20d^*^
+ IR-04 24 mg/kg	517	103.4 ± 0.81^d*^	511	102.20 ± 0.70^d*^
+ IR-04 48 mg/kg	526	105.20 ± 1.15^d*^	522	104.40 ± 0.87^d*^

SE: Standard error of the mean; NC: Negative control; DOX: Doxorubicin group; CIS: Cisplatin group; CYC: Cyclophosphamide group. ^a^Statistically compared to the NC group; ^b^Statistically compared to the DOX group; ^c^Statistically compared to the CIS group; ^d^Statistically compared to the CYC group; ^*^statistically significant difference (*p*<0.05; ANOVA/*t*-Student).

#### Splenic phagocytosis assay

Only the highest dose of IR-04 increased (*p*<0.05) the extent of splenic phagocytosis (1.66x). When this compound was combined with the commercial chemotherapeutic agents, there was a significant decrease in the splenic phagocytosis for all doses and all combinations. For the combination with doxorubicin, the reductions were 65, 49.44 and 35.28 percentage points for the doses of 12, 24 and 48 mg/kg, respectively. The same was observed for the combination with cisplatin, with reductions of 44.27, 25.16 and 11.15 percentage points, respectively; for cyclophosphamide, the corresponding reductions were 31.13, 29.56 and 25.47 percentage points (Table 4).

**Table 4:** Results related to splenic phagocytosis evaluation.

Experimental Groups	Phagocytosis	
	Absolute values	Mean ± SE
LOT 1		
NC	140	28.0 ± 2.55
IR-04 12 mg/kg	136	27.2 ± 2.01^a^
IR-04 24 mg/kg	140	28.0 ± 1.64^a^
IR-04 48 mg/kg	232	46.4 ± 1.03^a*^
LOT 2		
DOX	288	72.0 ± 1.08^a*^
+ IR-04 12 mg/kg	101	25.2 ± 0.75^b*^
+ IR-04 24 mg/kg	182	36.4 ± 1.16^b*^
+ IR-04 48 mg/kg	233	46.6 ± 1.20^b*^
LOT 3		
CIS	314	62.8 ± 0.96^a*^
+ IR-04 12 mg/kg	175	35.0 ± 1.87^c*^
+ IR-04 24 mg/kg	235	47.0 ± 2.21^c*^
+ IR-04 48 mg/kg	279	55.8 ± 1.65^c*^
LOT 4		
CYC	318	63.6 ± 1.32^a*^
+ IR-04 12 mg/kg	219	43.8 ± 1.65^d*^
+ IR-04 24 mg/kg	224	44.8 ± 1.46^d*^
+ IR-04 48 mg/kg	237	47.4 ± 0.67^d*^

SE: Standard error of the mean; NC: Negative control; DOX: Doxorubicin group; CIS: Cisplatin group; CYC: Cyclophosphamide group. ^a^Statistically compared to the NC group; ^b^Statistically compared to the DOX group; ^c^Statistically compared to the CIS group; ^d^Statistically compared to the CYC group; ^*^statistically significant difference (*p*<0.05; ANOVA/*t*-Student).

## Discussion

IR-04 is a novel compound whose toxicogenic effects are unknown. Thus, the present study is the first to report that this 4-aminoantipyrine derivative is not genotoxic.

For the isolated administration of this compound in the comet assay, the proportion of comets was lower in the IR-04-treated groups than in the control group. This property should be further explored in future studies because this ability to prevent basal genotoxic damage may be required when searching for chemoprotective agents because compounds that have antioxidant properties generally also have chemoprotective activity (Mantovani *et al.*, 2008; Bacanli *et al.*, 2015). This chemoprotective effect may have occurred because the molecule that was developed for the present study contains 4-aminoantipyrine, which has previously shown to have antioxidant activity. Moreover, 4-aminoantipyrine can inhibit the formation of free radicals, which can cause DNA damage (Jain *et al.*, 2006).

Another noteworthy characteristic of IR-04 is that, when administered alone, it can induce cell death in the liver and kidneys. Commercial anticancer drugs increase DNA damage, and therefore induce apoptosis in cancer cells (Brown and Attardi, 2005). However, normal cells can also acquire genomic/genetic lesions. Thus, compounds like IR-04 that are capable of inducing apoptosis without causing genotoxic or mutagenic damage are essential in the search for new chemotherapeutic drugs (Almeida *et al.*, 2005).

The designed molecule, IR-04, has the radical 1,4-dioxo-2-butenyl, which has been reported to have cytotoxic and anticancer activities (Jha *et al.*, 2010). Thus, some characteristics of this radical are desired in chemotherapeutic and/or cytotoxic compounds. This type of compound can induce DNA damage, making it a promising regulator of the cell cycle and leading to the elimination of damaged cells, including tumor cells.

A further confirmation that this compound does not cause genetic damage and toxicity is the fact that the treatment with the two lowest doses tested or with the low stimulation at the highest dose did not induce splenic phagocytosis in the animals. As reported in the literature, toxic compounds cause DNA damage and/or cell death through different mechanisms, and damaged cells, cell debris, and especially cells infected by viruses and bacteria are removed from the circulation by splenic phagocytosis (Ishii *et al.*, 2011). Therefore, the absence of splenic phagocytosis, as observed in the present study, indicates the lack of toxicity and/or toxicogenic damage.

The combination of IR-04 with commercial chemotherapeutic agents showed that it has antigenotoxic and prevents cell death, in addition to its ability to decrease splenic phagocytosis. The hypothesized chemopreventive characteristic of the compound could explain these data. Due to its chemopreventive nature, the compound could be used to prevent DNA damage, especially chemically induced damage; thus, the proportion of cells with DNA damage that should be removed from circulation would be lower.

When IR-04 is combined with the commercial chemotherapeutic agents doxorubicin, cisplatin and cyclophosphamide, it is capable of preventing DNA damage, cell death, and decrease of splenic phagocytosis. Thus, the combination with the tested commercial chemotherapeutic agents is discouraged. This suggestion is based on all the data indicating a reduction in the extent of genetic damage, which is one of the main modes of action of these drugs (Goldstein and Kastan, 2015), and which would lead to apoptosis, the desired anticancer effect (Ishii *et al.*, 2011).

Although the use of IR-04 is discouraged in combination with doxorubicin, cisplatin, or cyclophosphamide as a coadjuvant in anticancer treatment, when we analyzed just the induction of DNA damage that generates apoptosis, we could observe one of the desired anticancer effects. On the other hand, even though observing a decrease in DNA damage (antigenotoxic effect, reported in this study) we need to consider that cancer cells can be more susceptible than their normal correspondents when exposed to anti-cancer drugs (Oberley and Buettner, 1979; Cebrian *et al.*, 2006). Such difference is due, for example, to tumor cells containing less antioxidant enzymes, as is the case for superoxide dismutate, GSH peroxidase, and GSH reductase (Oliveira *et al.*, 2018).

According to this point of view, the antigenotoxic effect can be assumed as positive/beneficial for normal cells that are being affected by non-selective chemotherapeutic medicines. Moreover, it can be assumed that the reduction of genotoxic damage observed is associated with the capacity of IR-04 to initiate cellular cycle arrest. Future studies will be necessary to test this hypothesis. However, the literature reports that antioxidant, antigenotoxic, and/or chemopreventive compounds are capable to cause cell cycle arrest (Kaur *et al.*, 2006; Mölzer *et al.*, 2013; Srivastava *et al.*, 2016), and thus increase the cellular repair time, facilitating the repair of DNA damage and allowing to follow the regular cycle. In contrast, if the repair does not occur, or comes late, it leads the damaged cell to cellular death by apoptosis after the treatment with IR-04 in combination with doxorubicin, cisplatin, and cyclophosphamide.

Alkylating agents, such as cyclophosphamide, are capable of making covalent bonds with nucleophilic components such as the DNA. Thus, at the end of the process, the substitution of base pairs guanine-cytosine for adenine-thymine may occur. In addition, other mechanisms could generate cross-linking between two strands of DNA, resulting in breaks, such as the opening or removing guanine residues and/or alkylation of a second guanine. Thus, cyclophosphamide acts both on DNA synthesis and causes damage to the molecule (Hall and Tilby, 1992). Doxorubicin, an anthracycline antibiotic, is also capable of interfering with DNA transcription and replication because of its intercalating proprieties. Also, doxorubicin forms a tripartite complex with topoisomerase II and DNA, resulting in DNA breaks and apoptosis (Thorn *et al.*, 2011). Cisplatin, on the other hand, reacts with the DNA causing intra- and interfilament cross-links. These processes result in DNA strand break, coding error, and further apoptosis. In the present study, it was verified that IR-04 was able to reduce DNA damage caused by these three commercial chemotherapies that have different mechanisms of action (Dasari and Tchounwou, 2014). However, our results are not sufficient to predict how the IR-04 ester interfered in each mechanism of action and also presented a pattern of chemoprevention in combination with the chemotherapeutics. Our results are distinct from those presented by Oliveira *et al.* (2018). The authors evaluated the IR-01 acid, derived from the same pharmacophoric groups as IR-04, and did not find a pattern of chemopreventive response, which suggested interference in the mechanisms of action of the chemotherapeutic agents. Given the above, new studies regarding the mechanisms of action and evaluation of the combination of IR-01/IR-04 molecules with cyclophosphamide, cisplatin and doxorubicin are needed to clarify the effects of these compounds, particularly the induction of cell death.

In conclusion, IR-04 is an important compound in the search for anticancer drugs that have greater selectivity, because IR-04 induces cell death without using the DNA damage pathway. However, IR-04 should not be considered an adjuvant for chemotherapy in combination with doxorubicin, cisplatin, and cyclophosphamide due to the possibility of decreasing the genotoxic effects and cell death potential of these drugs.
